# Ethnobotanical Study of Medicinal Plants in Tehuledere District, Northwest Ethiopia

**DOI:** 10.1155/2024/3420352

**Published:** 2024-09-26

**Authors:** Worku Misganaw, Yalew Yiblet

**Affiliations:** ^1^ Department Biology Debark University, Debark, Ethiopia; ^2^ Department Biology Mekdela Amba University, Tulu Awuliya, Ethiopia

**Keywords:** conservation, ethnobotany, herbal treatments, medicinal plants, threats

## Abstract

Medicinal plants have constituted a fundamental aspect of human health and wellness for millennia. The objective of this study was to document medicinal plants used to treat human and livestock ailments in the Tehuledere District. The data were collected using semistructured interviews, focus group discussions, and field observations with local informants. Preference ranking, direct matrix ranking, and informant consensus factor were used for data analysis. A total of 63 medicinal plant species belonging to 59 genera and 41 families were documented. The predominant families identified were Lamiaceae and Asteraceae, each containing 6 and 5 species, respectively. Of the recorded medicinal plants, 53 (80.95%) species were used for human ailments, whereas 12 (19.05%) species were used for animal health issues. Among the recorded medicinal plant species, shrubs constitute the highest number with 35% species. The most frequently used plant parts were leaves, accounting for 59% of remedies targeting human ailments. The administration of herbal treatments was primarily oral, aimed at addressing various diseases. The most significant threat to these medicinal plants was attributed to agricultural expansion, followed by the collection of firewood. The aim of documenting the use of medicinal plants in the treatment of diseases was to capture traditional practices, with species such as *Eucalyptus globulus*, *Olea europaea* subsp. *cuspidata*, and *Lepidium sativum* serving as the foundational basis for future pharmacological studies. It is imperative to prioritize the conservation of *Laggera tomentosa* and *Urtica simensis* to safeguard biodiversity and the cultural traditions associated with these endangered species. Engaging local communities in the management and conservation of plant resources, along with the preservation of their traditional knowledge, presents a cost-effective and sustainable solution.

## 1. Introduction

Medicinal plants have constituted a fundamental aspect of human health and wellness for millennia. These plants have been used in traditional medical systems throughout the world by a variety of civilizations due to their bioactive components [1]. The knowledge reveals that specific plant-derived compounds (phytochemicals) can have beneficial therapeutic effects on the human body, helping to prevent and treat various diseases [2, 3]. In Ethiopia, modern medical systems coexist alongside the prevalent use of traditional medicine. The Ethiopian population primarily relies on herbal remedies derived from indigenous flora for the treatment of various ailments [4, 5]. The transmission of these practices across generations is facilitated by the oral sharing of knowledge among community members. Ethiopia's ethnic diversity, characterized by distinct languages and cultures, contributes to a vast array of medicinal plant applications [6].

The literature underscores the critical significance of medicinal plants in delivering primary health services in Ethiopia [7]. It is noteworthy that a considerable portion of the population, approximately 80% of humans and 90% of livestock, relies on traditional medicine to fulfill their healthcare requirements [8, 9]. The recent focus of ethnobotanical research has been to systematically document these practices [10]. Researchers use methodologies such as botanical identification, community surveys, and interviews with traditional healers to compile comprehensive databases of medicinal plants [6, 11]. This documentation is not only essential for the preservation of traditional knowledge but also crucial for future pharmacological research, which may facilitate novel therapeutic discoveries [12].

The escalating pressure on natural habitats resulting from agricultural expansion and urbanization presents considerable risks to the biodiversity of medicinal plants within Ethiopia [13]. The preservation of these invaluable resources for future generations is significantly reliant on conservation initiatives [14]. Challenges such as climate change, habitat loss, and socioeconomic issues endanger both the availability of therapeutic plants and the transfer of traditional knowledge, despite the rich ethnobotanical history [15, 16]. There is an urgent need for policies that support both the integration of traditional medicine into national healthcare systems and conservation efforts [17].

Ethiopia's oral traditions of traditional medicinal plants are largely passed down through generations, resulting in a significant gap in documentation and records, unlike formal education systems that rely on written texts [18, 19]. This transfer of knowledge occurs within families, communities, and traditional healers, who have ancient wisdom and practices [20]. This document gap not only limits the accessibility of indigenous knowledge but also impedes scientific research and validation of traditional medicinal practices [19].

The population of the Amhara region, particularly in the Tehuledere District, places significant reliance on medicinal plants due to their high cultural acceptability, affordability, and effectiveness in addressing specific health issues for both humans and livestock. However, the medicinal plants and indigenous knowledge within the Tehuledere District are under threat due to agricultural expansion, deforestation, and inadequate documentation of traditional plant knowledge. While a previous study [21] addressed the plants used in traditional medicine within the study district, it did not provide insights into how knowledge is transferred, comparisons of remedial plant knowledge among different social groups, rankings of medicinal plants based on their curative potential, threats to the plant resource base, and the current conservation status of these plants. Further ethnobotanical investigations are essential for the documentation of indigenous medical knowledge within the country. Therefore, the current study aims to record traditional medicinal knowledge and compile a comprehensive list of medicinal plants employed in the treatment of diseases affecting both humans and animals in the Tehuledere District.

## 2. Materials and Methods

### 2.1. Description of the Study Area

**Figure 1:**
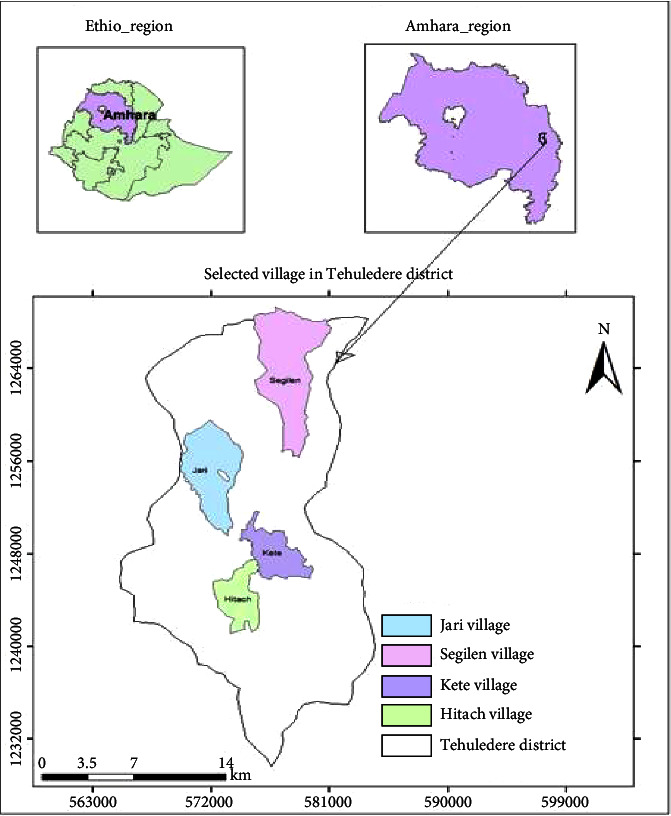
Map of the study area with selected kebele (Tehuledere District).

The Tehuledere District is located between 11°10′30″ N–11°29′25″ N latitude and 39°35′30″ E–39° 45 45 E longitude (Figure 1). Hayq is the main town in the district. It is located 430 km from Addis Ababa in the northeast direction and 460 km from Bahir Dar Town. The Tehuledere District has 21 administrative villages situated in three different agroecological zones, i.e., Kola, Woina Dega, and Dega. The altitude of Tehuledere ranges from 500 m.a.s.l. along the boundary with the South Wollo Zone to 2700 m.a.s.l. along its southwest border.

The district is classified into three agroclimatic zones according to the conventional agroecological categorization method, which takes into account temperature and altitude. These are called Kola/warm semiarid/lowland (500–1500 m), Woina Dega/cool subhumid/middle land (1500–2300 m.a.s.l.), and Dega/cool to humid/highland (2300–3200 m.a.s.l.) [22, 23]. According to the Rural Development and Agriculture Office Report, the Kola agroclimatic zone makes up 13.8% of the total area of the district, whereas the Woina Dega and Dega agroclimatic zones make up 70.6% and 15.6%, respectively. High and lowland climates are marked by extreme weather, whereas areas in the medium altitude range experience pleasant weather [24]. The Tehuledere Rural Development and Agriculture Office reported that the district's soil covers 15% black soils (vertisols), 40% red soils (nitosols and acrisols), 10% brown soils (cambisols and luvisols), and 35% gray soils (fluvisols and gleysols), and agriculture is the most common source of income for 75% of the population [24, 25].

The Tehuledere District in the Amhara Region of Ethiopia has a total population of 117,877 people, with a decrease of 1.14% from the 1994 census, according to the 2007 national census by the Central Statistical Agency (CSA) of Ethiopia. The district has 59,300 males and 58,577 females, with 14,745 (12.51%) living in urban areas. The population density of Tehuledere is 290.79 people per square kilometer, which is higher than the zonal average of 147.58 and the regional average of 405.37 people per square kilometer. The district has a total of 28,780 households, with an average of 4.1 people per family, and 27,643 dwelling units. The majority of the population (90.43%) identifies as Muslims, whereas 9.35% are followers of Ethiopian Orthodox Christianity.

### 2.2. Climate

**Figure 2:**
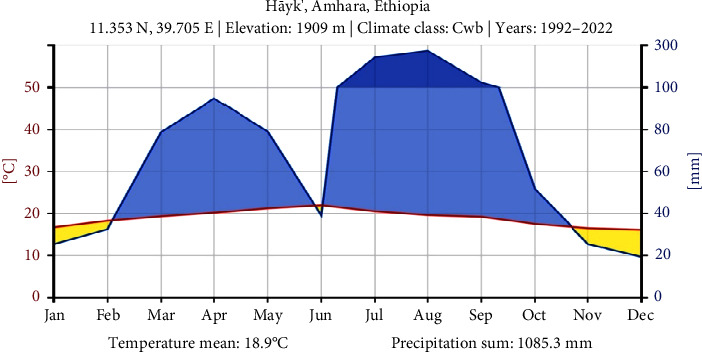
Climadiagram of the Tehuledere District data from 1992 to 2022 (*Source:* ClimateCharts.net [26]).

Studies of 30 years of supply of climate data (1992–2022) from Hayk Station using the interactive web platform Climate Charts.net for climate analysis revealed a bimodal pattern of rainfall distribution in the *International Journal of Digital Earth* [26], with the highest amounts occurring between March and May and July and October. The study area recorded a mean annual rainfall of 1085.3 mm, indicating the region's average level of precipitation throughout the year. The study analyzed rainfall and mean annual temperature, revealing an average of 18.9°C, providing valuable information on the climatic conditions in the study area (see Figure 2).

### 2.3. Vegetation

The study area is located in the evergreen dry habitat of Afromontane, known for its unique ecological characteristics and plant composition, with several notable plant species identified. According to Masresha and Melkamu [27], *Olea europaea* subsp. *cuspidata*, *Juniperus procera*, *Carissa spinarum*, and *Rosa abyssinica* are the characteristic feature plants of the dry evergreen Afromontane vegetation. The study area also includes various shrubs, herbs, and tree species, such as *Eucalyptus globulus* (introduced), *Juniperus procera*, *Vachellia abyssinica*, *and Olea europaea* subsp. *cuspidata*, and *Hesperocyparis lusitanica*, in the Mofa Forest of mountainous regions [28, 29].

### 2.4. Selection of Study Sites

A reconnaissance survey was conducted on September 15 and October 30, 2023, to evaluate the study area. Based on the local agroecological zones, four study sites were chosen: Hitach (Dega/cool to humid/highland), Kete (Woina Dega/cool subhumid/middle land), and Jari and Segilen (Kola/warm semiarid/lowland), as suggested by elders and local authorities in the Tehuledere District, taking into account the accessibility of traditional medicinal practices.

### 2.5. Sample Size and Informants' Selection

The sample size for the proposed study was determined using Cochran's sample size formula, as recommended by Kotrlik and Higgins [30], as follows:

(1) $$\begin{array}{c} N=\frac{N}{1+N{\left(e\right)}^{2}}, \end{array}$$

where *N* = total number of households in the district, *e* = maximum variability of making error at 95% confidence level (0.1 or ±10%), and 1 = the probability of the event occurring.

From the representative villages, a total of 120 informants (80 men and 40 women) were chosen at random in the selected villages, and the distribution of general and key informants is shown in Table 1. According to Giday et al. [31], general informants and knowledgeable traditional medicine practitioners (key informants) of the Tehuledere District were chosen by random and purposive sampling techniques, respectively. Twenty key informants were meticulously and purposefully selected based on recommendations from local experts, representatives of the local government, and wise elders who possess extensive knowledge in the field. In the process of selecting key informants, careful attention was paid to the quality and merit of the justifications provided by certain participants during interviews. This additional factor was taken into account to ensure the inclusion of highly qualified and knowledgeable individuals as key informants. As they are traditional specialists and custodians of indigenous knowledge about therapeutic herbs, local healers were immediately recognized as key informants.

For FGDs, 12 participants (8 males and 4 females) were selected from the designated villages in Tehuledere District using purposive sampling. The participants included elderly people and traditional healers who had specific knowledge and experiences with traditional medicines. The FGDs were conducted in two separate groups, with male participants in one group and female participants in the other, to facilitate open and in-depth discussions within each homogeneous group. The FGD sessions were held at a convenient location within the community and were facilitated by the researcher with detailed note-taking used to document the discussions for subsequent analysis.

### 2.6. Ethnobotanical Data Collection

The ethnobotanical data collection was carried out in the field from November 20 to January 30, 2023. Throughout the study, a combination of semistructured interview questionnaires, field observations, and group discussions served as the primary methods employed to collect data. Consistent with the study objective, a set of questionnaires was formulated in English and subsequently translated into the Amharic language, which is the local language spoken in the study area. This linguistic adaptation aimed to ensure effective communication and comprehension between study participants, facilitating their meaningful contribution to the investigation. The interview included details about the vernacular names of medicinal plants, the diseases they treat, the parts of the plant that are used, preparation and administration techniques, conservation measures, and other uses. In addition to semistructured interviews, group discussions and guided field trips with key informants were used to collect data. Four to five key informants participated in conservation strategies, challenges of medicinal plants, and the transmission and preservation of knowledge from one generation to the next. The voucher samples were collected through field observation at the study sites and in home gardens.

### 2.7. Collection and Identification of Plant Specimens

Voucher specimens of potentially medicinal plants were collected during field observation. The plant samples were then dried and pressed to facilitate identification. Information on the identification of the pressed sample specimens was available in the Mekdela Amba University Little Herbarium, as well as in the field. The World Flora Online Plant List [32] was used to confirm the botanical names and families of the reported medicinal plant species after the collected plant samples were brought to botanical experts [33].

### 2.8. Data Analysis

The analysis conducted in this study used descriptive statistical techniques to evaluate data pertaining to medicinal plants, with a particular emphasis on local knowledge. Quantitative descriptions were employed to interpret and generalize the findings, using Microsoft Excel and R software version 4.4.1 for analytical purposes. The results of the study are presented in the form of tables and graphs.

#### 2.8.1. Preference Ranking

Following Abebe and Chane Teferi [34], a preference ranking method was used for selecting the six most significant medicinal herbs used to treat tonsillitis. The goal of this exercise was to determine which medicinal herbs were best for treating tonsillitis, using ten randomly chosen informants. To ensure a comprehensive assessment of the efficacy of medicinal plants, informants received plants and clear instructions to classify them according to their perceived effectiveness. The classification system involved assigning higher ranks to the most valued plant species (6) and lower ranks to the least valued plant species (1). This ranking approach allowed the identification of the most preferred and highly regarded medicinal plants within the community, providing valuable information on local perceptions of efficacy.

#### 2.8.2. Direct Matrix Ranking

To evaluate the multipurpose usage of different plant species, a direct matrix ranking approach was used. This involved gathering information from informants and selecting eight specific plant species that the community used extensively for various purposes. Ten key informants were selected to assign use values to each attribute. Medicine, food, fodder, firewood, charcoal, fencing, and construction are among the use values. The mean values for each species were added and ranked according to the data collected from the respondents.

#### 2.8.3. Informant Consensus Factor (ICF)

To assess the level of consensus among informants about reported cures for a specific group of diseases, the ICF was calculated for each category [35]. This factor helps determine the degree of agreement or consensus among the informants on the use of medicinal plants to treat the listed diseases. The formula for ICF is as follows:

(2) $$\begin{array}{c} \text{ICF}=\frac{\left(\text{Nur}-\text{Nt}\right)}{\left(\text{Nur}-1\right)}, \end{array}$$

where Nur = number of use citations for a specific disease category and Nt = number of taxa used to treat the specific disease category.

## 3. Results

### 3.1. Sociodemographic Status of Informants

The majority of the informants (37.5%) belonged to the 60–70 age group, followed by those in the 40–50 age group (30%) and the 30–40 age group (20.8%). This indicates that traditional knowledge regarding medicinal plants is primarily retained by the older generation. Additionally, male informants (66.7%) significantly outnumbered their female counterparts (33.3%). A significant proportion of the informants (68.3%) were found to be illiterate, whereas only 31.7% possessed literacy skills. The majority of the informants (83.3%) was categorized as general informants, whereas 16.7% were classified as key informants (Table 2).

### 3.2. Diversity of Medicinal Plant Species

The current study documented a total of 63 different species of medicinal plants distributed in 59 genera and 41 families that have been recorded in the study area (Tables 3 and 4). In the district, with respect to the diversity of medicinal plant species, the highest numbers of medicinal plant species were reported in the Lamiaceae families, contributing 6 species, and Asteraceae 5 species, followed by Fabaceae and Solanaceae, contributing 4 species each.

### 3.3. Growth Form of Medicinal Plants Used to Treat Human Ailments

**Figure 3:**
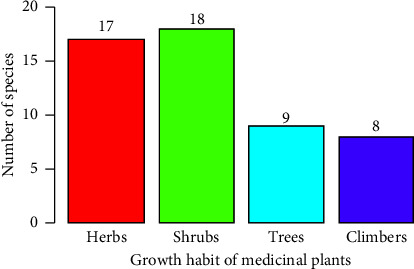
Growth habit of medicinal plants used to treat human ailments.

In the study area, the growth habit of medicinal plants in the Tehuledere District was predominantly composed of shrubs, comprising 18 (35%) species, followed by herbs, comprising 17 (33%) species, and trees, comprising 9 (18%) species (Figure 3). This result indicates that shrubs are the most commonly used plant growth habit in the traditional treatment of human ailments within the study area.

### 3.4. Plant Medicinal Parts Used to Treat Human Disease

**Figure 4:**
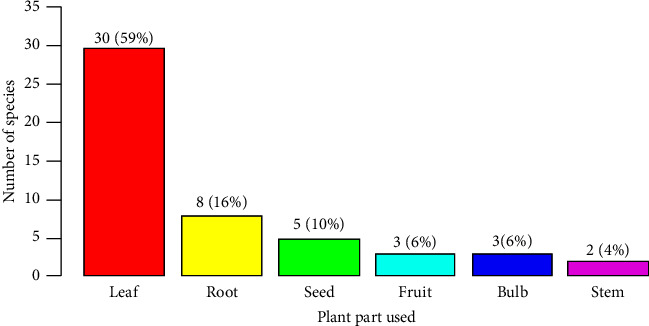
Parts of plants used for human remedy preparation.

In the Tehuledere District, various plant parts such as leaves, roots, seeds, stems, barks, and fruits are commonly employed in the treatment of human health issues. Among these parts of the plant, leaves were the most used, accounting for 30 (59%) followed by roots that comprised 8 (16%) species and seeds that comprised 5 (10%) species. This result shows the extensive use of leaves as a primary plant part in the preparation of remedies to address human health problems (Figure 4).

#### 3.4.1. Mode of Preparation Used to Treat Human Ailments

In the study area, various techniques were used in the preparation of medicinal plants. The most prevalent method was crushing, which was used in the preparation of 10 (20%) species, followed by pounding, used for 9 (18%) species, and squeezing, used for 8 (16%) species (Table 5).

#### 3.4.2. Route of Administration

**Figure 5:**
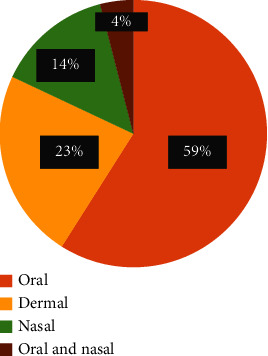
Route of administration of medicinal plants used to treat human ailments in the study area.

In the study area, the main method of administration was oral, accounting for 59% of all instances, followed by dermal administration, which constituted 23% of all occurrences (Figure 5).

#### 3.4.3. Dosage and Unit of Measurement

Different tools, including a handful (“*Efeign*”), glass (“*Birchiko”*), cup (“*Sini”*), and spoon (“*Mankia*”), were used to determine dosages. Dosages can be influenced by various factors, including individual age. Children, adults, and elderly individuals may require different dosages due to differences in body weight, metabolism, and physiological characteristics. Traditional medicine systems often take these factors into account and may have guidelines or traditional knowledge regarding dosage adjustments for different age groups (Figure 5).

### 3.5. Grown Forms of Medicinal Plants Used to Treat Animal Ailments

**Figure 6:**
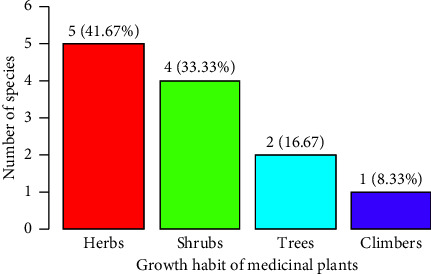
Habit of medicinal plants used to treat animal ailments.

The field study investigated the use of 12 species of medicinal plants to treat diseases in livestock within the study area. The growth form of these medicinal plants for livestock was predominantly shrubs, accounting for 5 (41.67%) species, followed by herbs with 4 (33.33%) species and trees with 2 (16.67%) species (Figure 6).

#### 3.5.1. Plant Part Used to Treat Animal Diseases

**Figure 7:**
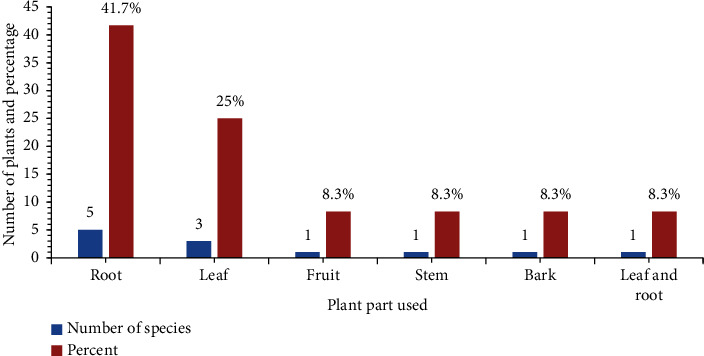
Part used by medicinal plants to treat animal diseases in the study area.

The study found that when using medicinal plants to treat diseases in cattle, the root component of the plant was used more frequently compared with other plant parts. Roots were used in the preparation of 5 (41.7%) species, whereas leaves were used in the preparation of 3 (25%) species (Figure 7).

#### 3.5.2. Route of Administration

**Figure 8:**
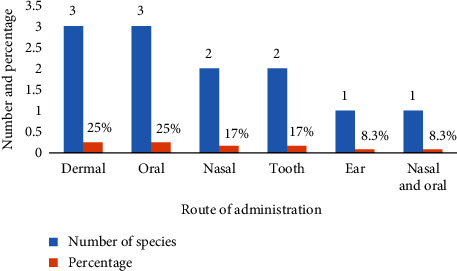
Application routes of medicinal plants used to treat animal ailments in the study area.

A range of application methods, such as dermal, oral, nasal, and dental administration, have been used to treat ailments in animals. The oral and dermal routes constituted 25% of the medications examined in this study (Figure 8).

### 3.6. Ranking of the Most Important Medicines

#### 3.6.1. Preference Ranking

In the preference ranking exercise, *Schinus molle* was considered the most effective herbal remedy for the treatment of tonsillitis, whereas *Ocimum lamiifolium* was ranked as the least effective (Table 6).

#### 3.6.2. Direct Matrix Ranking

*Eucalyptus globulus* placed highest in the direct matrix ranking findings, indicating that it is the most commonly used plant among the local community for a variety of purposes. The second was *Olea europaea* subsp. *cuspidata*; the third was *Cordia africana*; and the last was *Vachellia abyssinica* (Table 7).

#### 3.6.3. ICFs

Public health issues (for which the informants offered treatments and declared themselves cured) were divided into nine disease categories, and the degree to which the informants agreed with their treatments was evaluated. The categories of psychiatric disorders (ICF = 0.68), respiratory organs and throat (ICF = 0.65), abdominal and gastrointestinal problems (ICF = 0.61), and acute diseases (ICF = 0.58) showed comparatively stronger informant agreements (Table 8).

### 3.7. Threats and Conservation Practice of Medicinal Plants

#### 3.7.1. Threats to Medicinal Plants

The study indicated that human activities, including agricultural expansion, firewood collection, construction, drought, and urbanization, present significant threats to medicinal plants, with agricultural expansion identified as the most severe threat (Table 9).

#### 3.7.2. Conservation Practice of Medicinal Plants

The study identified several factors, both anthropogenic and natural, that are threatening the survival of medicinal plants in the Tehuledere District. As natural vegetation is increasingly destroyed, the habitats of these vital medicinal plants are becoming progressively threatened. The rural community relies on these plants for various aspects of their livelihoods. Interviews with informants revealed that the primary threats to medicinal plants include agricultural expansion, construction activities, and drought, which are contributing to the decline of natural vegetation. These findings highlight the urgent need for conservation efforts to protect the valuable medicinal plant resources in the study area.

## 4. Discussion

### 4.1. Sociodemographic Features of Informants

Among informant groups, older individuals (age groups > 70) continue to possess more indigenous and local knowledge about the use of medicinal plants than younger individuals (age groups < 40). Similar findings were also documented by Siraj, Belew, and Suleman [36], Bekele et al. [37], Kidane, Gebremedhin, and Beyene [38], and Lulekal et al. [39] in various regions of the country, indicating that older informants possess superior ethnobotanical knowledge compared with younger ones. Additionally, the study revealed a noticeable disparity in the knowledge of medicinal plants shared by traditional healers, with females demonstrating more comprehensive knowledge compared with their male counterparts within the study district. The traditional knowledge and use of medicinal plants are primarily concentrated among the older generation and female traditional healers, which may have implications for the preservation and transmission of this valuable ethnobotanical knowledge in the future.

### 4.2. Diversity of Medicinal Plants

In the Tehuledere District, a total of 63 species of medicinal plants have been documented. This figure surpasses those found in comparable ethnobotanical studies conducted in Ethiopia. For instance, the Babile District noted 51 species [40], the Gimbi District identified 49 species [41], and the Gemad District mentioned 31 species [42]. This is due to the extensive use of medicinal plants in the Tehuledere District, which can be attributed to several factors. These include the absence of formal healthcare options, a heightened cultural reliance on traditional medicine, and the relatively low cost associated with the plants used in traditional medicinal practices. The presence of a greater number of medicinal plants (*n* = 63) for the treatment of human diseases in the Tehuledere District may be attributed to the high prevalence of various human ailments, including cough, abdominal pain, malaria, tonsillitis, diarrhea, the evil eye, and wounds. This finding aligns with results from other studies conducted in Ethiopia [43, 44], which indicate that a greater number of plant species have been used to treat human ailments.

### 4.3. Growth Forms of Medicinal Plants

In the Tehuledere District, shrubs represent the predominant plant form used for remedies pertaining to human ailments, comprising a significant portion of the medicinal plants employed in the study area. This trend may be attributed to the prevalent growth patterns observed in the region. Conversely, herbs emerged as the most frequently used medicinal plants for treating animal ailments in the current study, reflecting a greater relative abundance of herbs compared with other growth forms [45]. Similar findings have been documented in other regions of Ethiopia, where herbs are also recognized as the most commonly employed growth forms [16, 46]. This indicates reliance among the local population on shrubs and herbs, which are readily accessible.

### 4.4. Plant Parts Used to Treat Human and Livestock Ailments

The findings from the Tehuledere District reveal an interesting contrast in the use of different plant parts for the preparation of traditional remedies. Notably, the study determined that leaves are the most widely used plant part for treating human diseases in the district, a finding that is consistent with numerous other studies conducted in various regions of Ethiopia [47]. There are several reasons that can explain the predominance of leaves in the formulation of human health remedies. As indicated by Bekele et al. [37], leaves are often preferred due to their relative ease of preparation and the potential therapeutic benefits they offer compared with other plant parts. Leaves are generally more accessible, easier to harvest, and may contain a higher concentration of medicinally active compounds, making them a practical choice for traditional medicine preparation. It is noteworthy that the findings from the Tehuledere District differ from the report by Berhanu et al. [48], who found that fruits were frequently used in the Ambo District for traditional medicine preparation. This highlights the importance of investigating medicinal plant usage across different regions, as the preferences and practices may vary based on the local context and traditional knowledge.

In contrast, the study found that in the Tehuledere District, people regularly use roots to prepare remedies for treating animal diseases. This observation is corroborated by a similar study conducted in the Berbere District of Ethiopia, which also identified roots as the most frequently harvested plant part [49]. This suggests that the preference for root-based remedies may be a common practice in certain traditional medicine systems within the country for addressing animal health concerns. The use of roots for animal disease treatment can be attributed to factors such as the accessibility and availability of roots, as well as the potential concentration of bioactive compounds in the root tissues. However, the reliance on roots may raise sustainability concerns, as the harvesting of roots can be more damaging to the individual plant's survival compared with the harvesting of leaves or other aboveground plant parts.

In general, the contrast between the use of leaves for human disease treatment and roots for animal disease treatment underscores the need for a comprehensive understanding of the local context and traditional knowledge when studying the use of medicinal plants. These insights can inform the development of sustainable harvesting practices and conservation strategies to ensure the continued availability of these valuable natural resources.

### 4.5. Method of Preparation and Route of Administration

The study district used various methods for the preparation of remedies, with the crushing technique being the most effective, consistent with the findings of the previous study findings [14] conducted in Kofele District, West Arsi Zone, Oromia regional state, Ethiopia. As scholars [50] indicated, traditional healers believe that powdering improves the extraction of bioactive components, enhancing the efficacy of medications against a variety of human diseases. Oral administration is the most popular method of treating human ailments due to its favorable environment for a rapid physiological reaction against pathogens. This result is aligned with the studies [38] reported in Ganta Afeshum District, Northern Ethiopia. Kassa, Asfaw, and Demissew [51] reported that oral administration offers a unique advantage for traditional medicine practitioners, allowing them to prevent complications during antidote treatment.

### 4.6. Dosage and Unit of Measurement

The study identified various tools for determining dosages of traditional medicines, including a handful (“*Efeign”*), a glass (“*Birchiko”*), a cup (“*Sini”*), and a spoon (“*Mankia”*). Similar work was conducted in [52] in Ensaro District, Ethiopia. The study found that the dosage of traditional medications can be influenced by an individual's age, highlighting the need to consider age-related factors. Amsalu et al. [53] indicate that inconsistency and lack of standardization in dosages may be detrimental factors to the use and acceptance of traditional herbal medicine.

### 4.7. Ranking of the Most Important Medicinal Plants

The result indicated that psychiatric disorders possess the highest ICF value, signifying that the plants are widely recognized and frequently used within the community for particular health issues. Moreover, there exists a significant cultural importance associated with the use of these plants, fostering a collective comprehension of their advantages. This finding is correlated with the other studies [51] reported in Sheka Zone of Southern Nations Nationalities and Peoples Regional State, Ethiopia. The high value of the ICF reflects the popularity and effectiveness of these plant-based treatments among informants. The study preference ranking found that *Schinus molle* L. was the most effective plant species for treating tonsillitis in the local community, while *Ocimum lamiifolium* Hochst. was ranked the least effective, indicating a preference for this herbal remedy. The analysis of this finding corresponds with a previous scenario [54] in Ameya District, Ethiopia. Based on the findings of the study, it was found that *Eucalyptus globulus* Labill. was identified as the most preferred multipurpose medicinal plant in the study area. Tadesse and Teka [54] reported similar results in their study area. This suggests that the local community highly values the medicinal properties and diverse uses of this plant.

### 4.8. Threats and Conservation Practice of Medicinal Plants

As natural vegetation is diminished, the habitats of these vital medicinal plants are increasingly endangered. The medicinal plant species *Laggera tomentosa* and *Urtica simensis* were listed on the IUCN Red List of Threatened Species [55, 56]. The findings of this study are consistent with those documented by Masresha in the flora of Simien Mountains National Park, located in North Gondar, northwestern Ethiopia [57]. Due to habitat destruction and excessive harvesting, numerous medicinal plants are facing significant threats to their survival. This study indicates that anthropogenic factors, such as agricultural development, construction, drought, and the collection of firewood, have all contributed to the decline of medicinal plant species. The loss of access to medicinal plants has been caused by agricultural expansion. Similar findings were observed in various studies regarding the threats to medicinal plants in Gozamin District [58], Gera District [59], and Heban Arsi District, Oromia, southeastern Ethiopia [60]. It has been observed that the efforts of local residents to conserve the medicinal plants of the district have been woefully inadequate. However, some traditional practitioners have initiated the preservation of medicinal plants by cultivating them in home gardens.

### 4.9. Limitation of the Study

The exploration of antibacterial, anti-inflammatory, and antioxidant activity in medicinal plants was hindered by a lack of funding and a poorly equipped laboratory.

## 5. Conclusions

The findings of this study reveal a substantial body of traditional knowledge within the Tehuledere District concerning the use of medicinal plants for the treatment of ailments affecting both humans and livestock. It can be concluded that shrubs represent the primary source of traditional remedies, followed by herbs. Moreover, it was noted that leaves are the most commonly used plant parts, with roots also being significant for the preparation of remedies for humans and livestock, respectively. The prevalent use of certain medicinal plants, along with their various parts and the dependence on fresh plant materials, poses potential threats to the sustainability of these medicinal plants in the study area. Factors such as agricultural expansion, firewood collection, construction activities, drought, and urbanization have been identified as major contributors to the decline of plant taxa. Therefore, the establishment of comprehensive awareness regarding the management and conservation of medicinal plants, as well as their habitats, which serve as the home for most species, is essential. Additionally, it is important to establish coordination between traditional healers and the district's health offices to ensure the effective provision of traditional medicines.

## Figures and Tables

**Table 1:** Distribution of general and key informants.

**Names of kebeles**	**General informants**		**Key informants**		**Total**
	**M**	**F**	**M**	**F**	
Jari	17	10	4	1	32
Segilen	17	8	3	2	30
Kete	11	7	2	3	23
Hitach	19	11	3	2	35
Total	100		20		120

**Table 2:** Demographic details of informants in the Tehuledere District.

**Sociodemographic feature**	**Category**	**Frequency**	**Percentage**
Age	30–40	25	20.8
	40–50	36	30
	60–70	45	37.5
	Above 70	14	11.7

Gender	Male	80	66.7
	Female	40	33.3

Education	Illiterate	82	68.3
	Literate	38	31.7

Informant category	Key informants	20	16.7
	General informants	100	83.3

**Table 3:** List of medicinal plants used for the treatment of livestock.

**Family**	**Scientific name**	**Local name**	**Hb**	**Habitat**	**Plant part used**	**Ailments**	**Method of preparation**	**RA**	**Voucher number**
Apiaceae	*Foeniculum vulgare* Mill	Kumen	H	Wild	Leaf	Devil disease	Leaf is pounded, squeezed, and then drunk for 2 days	Oral	WY04

Crassulaceae	*Kalanchoe petitiana* A. Rich	Andawula	H	Wild	Root	Ear disease	Root is crushed, squeezed, and applied a few drops to the ear	Ear	WY05

Lamiaceae	*Rotheca myricoides* (Hochst.) Steane & Mabb. dicolor (Klotzsch) Verdc.	Misirich	S	Wild	Root	Body swelling	The pound leaf mixes with little water and drank in a glass of water for 5 days	Oral	WY017

Meliaceae	*Melia azedarach* L.	Nim	T	HG	Bark	Toothache	The pounded bark is held by dressing the affected tooth for minutes	Tooth	WY021

Poaceae	*Arundo donax* L.	Shembeko	H	HG	Stem	Bone fracture	The stem is splintered into many fractions, and the affected part is tied until recovery	Dermal	WY028

Ranunculaceae	*Clematis simensis* Fresen.	Azoareg	C	Wild	Leaf and root	Wound	The root is pounded, powdered, mixed with butter, and applied to the affected part until recovery	Dermal	WY032

Salicaceae	*Dovyalis caffra* (Hook.f. & Harv.) Sim	Koshim	T	H G	Fruit	Dandruff	Fruits are crushed, squeezed, and rubbed on the affected part	Dermal	WY034

Scrophulariaceae	*Verbascum sinaiticum* Benth.	Qetetina	S	Wild	Root	Rh factor	Root is crushed and mixed with *Hordeum vulgare* seed pounded and given to the donkey at night	Oral	WY037

Solanaceae	*Nicotiana tabacum* L.	Tembaho	H	HG	Leaf	Toothache	Leaf is chewed and held by the infected teeth	Tooth	WY044
	*Withania somnifera* (L.) Dunal	Hidebuda	S	Wild	Root	Evil eye	Root is burned and smoked for 3–5 days at night time	Nasal and oral	WY046

Verbenaceae	*Lippia abyssinica* (Otto & A. Dietr.) Cufod. Walp.	Kessie	S	Wild	Leaf	Eye disease	Leaves are powdered, put on fire, and smoked for 3 days at night	Nasal	WY062

Abbreviations: C = climber, Hb = habit, HG = home garden, RA = route of administration, S = shrub, T = tree.

**Table 4:** List of medicinal plants used to treat human diseases.

**Family**	**Scientific name**	**Local name**	**Hb**	**Habitat**	**Plant part used**	**Ailments**	**Method of preparation**	**RA**	**Voucher number**
Asteraceae	*Artemisia abyssinica* Sch. Bip. ex A. Rich	Chikugn	H	Hg	Leaf	Common cold	The fresh leaf is inhaled through the nose for duration of one day	Oral	WY07

Acanthaceae	*Justicia schimperiana* T. Anderson	Sensl	S	Wild	Leaf	Hepatitis	Pounded dried leaves, mixed with a cup of tea, and then drunk in the morning	Oral	WY03

Amaranthaceae	*Amaranthus graecizans* L.	Aluma	H	Wild	Leaf	Headache	The leaf is crushed and then prepared in the form of a stew, traditionally eaten together with injera	Oral	WY08

Amaryllidaceae	*Allium sativum* L.	Nech Shinkurt	H	HG	Bulb	Malaria	Ground bulb is mixed with butter and drunk in glass water	Oral	WY01
	*Allium cepa* L.	Key shinkurt	S	HG	Bulb	Skin infection	The bulb of *Allium cepa*, along with the root of *Rumex abyssinicus*, is crushed and combined with the juice of *Citrus aurantifolia* and salt. The resulting mixture is then topically applied as a cream to the affected area	Dermal	WY010
	*Crinum abyssinicum* Hochst. ex A. Rich	Yejib Shinkurt	H	Wild	Bulb	Ear disease	Bulb is pounded and squeezed with water and then added a drop on the ear	Dermal	WY016

Anacardiaceae	*Schinus molle* L.	Qundoberberie	T	HG	Seed	Tonsillitis	Seeds are chewed slowly and taken with tea	Oral	WY012

Apocynaceae	*Carissa spinarum* L.	Agam	S	Wild	Leaf	Diarrhea	The powdered leaf is blended with coffee and subsequently boiled, after which one cup is consumed until recovery	Oral	WY02

Asparagaceae	*Asparagus africanus* Lam	Yeset Qest/Sereti	C	Wild	Leaf	Evil eye	The leaf is powdered and fumigated	Nasal	WY014

Asteraceae	*Guizotia scabra* Chiov.	Yedega Mech	H	Wild	Root	Epilepsy	A root is pounded, mixed with a cup of water, boiled, and drunk	Oral	WY019
	*Lactuca sativa* L.	Selata	H	HG	Leaf	Cough	Leaf is soaked in boiled milk and taken four successive days	Oral	WY011
	*Laggera tomentosa* (A. Rich.) Sch. Bip. ex Oliv. & Hiern ^∗∗^	Alashume	S	Wild	Leaf	Wound	The leaf is pounded and subsequently diluted with water, after which it is applied topically as a cream on the wound	Dermal	WY018
	*Tagetes minuta* L.	Yeahiya aruti (Gime)	H	Wild	Leaf	Dandruff wound	The leaves are ground, and the sap is collected and applied over the infected body	Dermal	WY020

Fabaceae	*Caesalpinia decapetala*	Kentafa	S	Wild	Root	Evil eye	The root is pounded and burned on fire, and the smoke is inhaled	Nasal	WY013

Asteraceae	*Vernonia amygdalina* Delile	Grawa	T	Wild	Root	Evil eye	The root is burned and sniffed smoke for 5–7 days during night	Nasal	WY023

Boraginaceae	*Cordia africana* Lam	Wanza	T	Wild	leaf	Common cold	Ground leaves are mixed with soil, water, and *Eucalyptus globulus*, boiled, and fumigated	Nasal	WY022

Brassicaceae	*Lepidium sativum* L.	Feto	H	Wild	Seed	Hypertension	The seeds are crushed into a fine powder and combined with *Allium sativum* and then eaten with injera	Oral	WY031

Cucurbitaceae	*Citrullus colocynthis* (L.) Schrad	Yemidir Embuy	C	Wild	Root	Colic pain	The ground root is mixed with honey and injera and then swallowed	Oral	WY035
	*Cucurbita pepo* L.	Duba	C	HG	Fruit	Headache	The leaf is crushed into a fine powder and securely fastened to your head with a gentle tie	Dermal	WY051
	*Zehneria minutiflora* (Cogn.) C. Jeffrey	Heregresa	C	Wild	Leaf	Febrile illness	The leaf is boiled, fumigated, and drunk, and also squeezed and mixed with a cup of coffee in the morning	Oral	WY041

Euphorbiaceae	*Ricinus communis* L.	Gulo	T	Hg	Fruit	Cancer	The fruit is pounded to a pulp and mixed with *Cyphostemma cyphopetalum*. This mixture is then applied by gently rubbing it on the injured part	Dermal	WY029

Fabaceae	*Caesalpinia decapetala*	Kentafa	S	Wild	Root	Evil eye	The root is pounded and burned on fire, and the smoke is inhaled	Nasal	WY024
	*Cicer arietinum* L.	Shimbra	H	Hg	Seed	Malaria	Seeds are mixed with a bulb of *Allium sativum* in water and consumed 1–2 cups every morning for 5 days	Oral	WY033
	*Lathyrus sativus* L.	Guaya	H	HG	Leaf	Leech	The leaf is gently crushed, which releases its aromatic essence, mixed with the gesho leaf, and drunk at midday	Oral	WY060
	*Vachellia abyssinica* (Hochst. ex Benth.) Kyal. & Boatwr	Girar	T	Wild	Root	Incantation	The roots of *V*. *abyssinica* and *Hydrangea arborescens* are combined and added to boiling water. The resulting steam is then inhaled through the mouth and nasal openings	Nasal and oral	WY059

Hypericaceae	*Hypericum revolutum* Vahl.	Amja	S	Wild	Leaf	Hypertension	The leaf is crushed and then mixed with lemon juice, and one cup of the mixture is drunk daily	Oral	WY048

Lamiaceae	*Leonotis ocymifolia* (Burm. f.) Iwarsson	Fereszeng	S	Wild	Root	Scorpion	Chew the root directly for a period of 7 days	Oral	WY042
	*Mentha spicata* L.	Nana	H	Wild	Leaf	Headache	The leaf is crushed, brought to a boil with water, and drunk like a tea	Oral	WY06
	*Ocimum lamiifolium* Hochst	Damakase	S	HG	Leaf	Febrile illness	The leaves are gently squeezed and infused into a cup of warm tea or coffee	Oral	WY09
	*Origanum majorana* L.	Ketenayu	S	Wild	Root	Kunchir	The root is crushed, squeezed, and rubbed on the affected part with cotton	Dermal	WY015
	*Rosmarinus officinalis* L.	Azmarino	S	HG	Leaf	Spider poison	The leaf is ashed, then combined with honey, and consumed for the duration of 5 days in the morning	Oral	WY026

Linaceae	*Linum usitatissimum* L.	Telba	H	Hg	Seed	Stomach pain	Seed is soaked in water for 1–2 days, and sugar is added and drunk.	Oral	WY030

Malvaceae	*Sida schimperiana* Hochst. ex A. Rich.	Chifrig	S	Wild	Leaf	Cough	The leaf is soaked in boiled milk and consumed in the morning for the duration of 3 days	Oral	WY055

Menispermaceae	*Stephania abyssinica* Walp.	Yayithareg	C	Wild	Leaf	Ear lesion	The leaf is squeezed and added through the ear by using cotton	Dermal	WY057

Musaceae	*Ensete ventricosum* (Welw.) Cheesman	Koba	S	HG	Root	Stomach complaints	Roots are pounded, mixed with water, boiled, and consumed as a drink	Oral	WY036

Myrtaceae	*Myrtus communis* L.	Adese	S	HG	Leaf	Dandruff	The leaves of *Myrtus communis* are crushed, combined with butter, and then applied topically to the affected area for a period of 3 days	Dermal	WY038
	*E*. *globulus* Labill	Bahirzaf/Nech	T	HG	Leaf	Febrile illness	The leaf is chopped and boiled with water, and the resulting solution is inhaled every morning for a period of 2 days	Oral	WY045

Oleaceae	*Jasminum grandiflorum* L.	Tembelel	C	Wild	Leaf	Infertility	Leaves of *Jasminum grandiflorum* are squeezed, and the pure liquid is drunk for 7 days	Oral	WY047
	*Olea europaea* subsp. *cuspidata* (Wall. & G. Don) Cif	Woira	T	Wild	Stem	Psychiatric disease	The stem is pounded, allowed to dry, burned, and then inhaled in the smoke	Nasal	WY054

Phytolaccaceae	*Phytolacca dodecandra* L'Hér.	Endod	S	HG	Leaf	Rabbis	The pounded leaf is mixed with *Coffea arabica*. Then, a cup of tea is drunk every morning and saved some for the evening	Oral	WY061

Poaceae	*Cymbopogon martini* (Roxb.) Will. Watson	Teji sar	H	Hg	Leaf	Devil	Leaves are burned and fumigated to remove “the devil” from at home	Oral and nasal	WY063

Polygonaceae	*Rumex nepalensis* Spreng	Tult	H	Wild	Leaf	Stomach ache	The leaf is squeezed and the juice is drunk with a spoon of honey for 3 days	Oral	WY039
	*Rumex nervosus* Vahl.	Embuacho	S	Wild	Leaf	Snake bite	The leaf is crushed, dissolved in water, and drank the resulting mixture for duration of 3 days	Oral	WY025

Rosaceae	*Rosa abyssinica* R. Br. ex Lindl.	Kega	S	Wild	Leaf	Skin disease	The leaf is squeezed and rubbed on the injured part	Dermal	WY027
	*Rubus apetalus* Poir.	Enjori	H	Wild	Leaf	Anemia	The leaf undergoes a process of boiling to extract its constituents and is combined with honey for added flavor and potential health benefits. Then, it was consumed regularly over a period of 1 week	*Oral*	WY049

Rubiaceae	*Galium spurium* subsp. *Spurium*	Chegogit (Asshect)	C	Wild	Leaf	Nasal bleeding	The leaves are squeezed, and the resulting juice or extract is inhaled through the nasal opening during bleeding	Nasal	WY056

Rutaceae	*Ruta chalepensis* L.	Chinadam	S	HG	Seed	Evil eye	The root of the plant is combined with *Allium sativum* and crushed liver of hyena. This mixture is then sniffed during times of sickness	Nasal	WY052

Salicaceae	*Salix mucronata* Thunb.	Ahaya/Riga	T	Wild	Stem	Gum disease	The stem is masticated and subsequently brushed until the sensation subsides	Oral	WY043

Sapindaceae	*Dodonaea viscosa* subsp. *angustifolia* (L.f.) J.G.West	Kitkita	T	Wild	Leaf	Skin rash	The leaf of *Dodonaea viscosa* subsp. *Angustifolia* is roasted, pounded, powdered, and mixed with butter and creamed in the affected part	Dermal	WY058

Solanaceae	*Datura stramonium* L.	Astenagirt or Bench	S	Wild	Leaf	Ear disease	The leaf is crushed and mixed with a glass of water. A spoonful of the solution is then administered through the ear in the morning for 3 days	Dermal	WY040
	*Physalis lagascae* Roem. & Schult.	Awut	C	Wild	Fruit	Hypertension	The fruit is peeled and then squeezed to extract the juice, which is collected and consumed daily	Oral	WY050

Urticaceae	*Urtica simensis* Hochst. ex A. Rich^∗∗^	Sama	H	Wild	Leaf	Gastritis	The young leaf is ground, cooked like a stew, and eaten with injera	Oral	WY055

Abbreviations: C = climber, Hb = habit, HG = home garden, RA = administration route, S = shrub, T = tree.

^∗∗^ = near threatened.

**Table 5:** Methods of preparation of medicinal plants in the study area.

**Method of preparation**	**Frequency**	**Percent (%)**
Crushing	10	20
Pounding	9	18
Powdering	3	6
Grinding	4	8
Squeezing	8	16
Inhaling	4	8
Boiling	6	12
Soaking	3	6
Chewing	2	4
Burning and fumigate	1	2

**Table 6:** Preference ranking of medicinal plants used to treat tonsillitis.

**Plant species**	**Respondents** **R**_1_–**R**_10_										**Total**	**Rank**
	**R1**	**R2**	**R3**	**R4**	**R5**	**R6**	**R7**	**R8**	**R9**	**R10**		
*Lepidium sativum* L.	3	1	2	5	5	5	4	6	3	4	38	5
*Schinus molle* L.	6	5	7	5	8	6	6	8	7	6	64	1
*Ocimum lamiifolium* Hochst.	5	6	7	5	8	5	6	7	7	6	62	2
*Citrullus colocynthis* (L.) Schrad.	6	5	7	5	4	5	6	7	7	8	60	3
*Zehneria minutiflora* (Cogn.) C. Jeffrey	6	5	5	7	5	6	3	4	2	8	51	4

**Table 7:** Results of direct matrix ranking of 6 species of multipurpose plants.

**Medicinal plants**	**Charcoal**	**Construction**	**Fencing**	**Fire wood**	**Fodder**	**Food**	**Medicine**	**Total**	**Rank**
*Vachellia abyssinica* (Hochst. ex Benth.) Kyal. & Boatwr. (Hochst. ex Benth.) Kyal. & Boatwr. (Hochst. ex Benth.) Kyal. & Boatwr.	2	2	3	4	3	0	4	21	7
*Carissa spinarum* L.	1	1	3	3	2	4	5	25	5
*Cordia africana* Lam.	2	5	3	4	3	2	3	31	3
*Eucalyptus globulus* Labill.	4	5	7	5	1	0	4	37	1
*Olea europaea subsp*. Cuspidata (Wall. & G. Don) Cif.	4	5	5	5	3	0	4	36	2
*Vernonia amygdalina* Delile	2	3	6	5	5	0	4	30	4

**Table 8:** Results of the informant's consensus factor (ICF) for human health problems.

**Category of disease**	**Nt**	**Nur**	**ICF**
Acute sickness (malaria, epilepsy, sudden illness, and psychiatric disease febrile illness)	9	20	0.58
Ailments associated with respiratory organs and throat/asthma, common cold, cough, bronchitis, and tonsillitis, sore throat	18	50	0.65
Animal nip (snakebite, antisnake venom, scorpion, and rabies)	5	7	0.33
Dermal diseases (scabies, skin disease, kunchir, skin cancer, tumor, dry skin, dandruff, wound, herpes, and skin rash)	21	29	0.29
Disease related to abdominal and gastrointestinal problems (stomach problem, intestinal parasite, ascariasis, gastritis and loss of appetite, dysentery and vomiting, and diarrhea)	22	55	0.61
Genitourinary problems (syphilis, gonorrhea, genital wart, and dysuria)	8	12	0.36
Organ diseases (liver problem, kidney problem, headache, existence of anal eye problem, ear parasite, and toothache)	22	45	0.52
Psychiatry problems (incantation, evil eye, epilepsy, and devil)	19	58	0.68
Reproductive system disorder (abnormal menstrual cycle, infertility, impotency, and others)	4	7	0.5

**Table 9:** Threats to medicinal plants in the study area.

**No.**	**Threatening factor**	**Informants**									
		**R**_1_	**R**_2_	**R**_3_	**R**_4_	**R**_5_	**R**_6_	**R**_7_	**R**_8_	**Total**	**Rank**
1	Agricultural expansion	7	6	6	7	6	6	7	7	52	1
2	Construction	7	3	6	7	6	4	3	7	43	3
3	Drought	5	5	5	5	5	5	4	3	42	4
4	Firewood	4	5	6	7	6	7	6	7	48	2
5	Urbanization	3	4	4	4	3	3	5	5	34	5

## Data Availability

All data are available in this article.
